# Implementing Exercise in Standard Cancer Care (Bizi Orain Hybrid Exercise Program): Protocol for a Randomized Controlled Trial

**DOI:** 10.2196/24835

**Published:** 2021-08-09

**Authors:** Maria Soledad Arietaleanizbeaskoa, Erreka Gil Rey, Nere Mendizabal Gallastegui, Arturo García-Álvarez, Ibon De La Fuente, Silvia Domínguez-Martinez, Susana Pablo, Aitor Coca, Borja Gutiérrez Santamaría, Gonzalo Grandes

**Affiliations:** 1 Biocruces Health Research Institute Barakaldo Spain; 2 Deusto University Bilbao Spain

**Keywords:** patients with cancer, physical activity, primary care, behavioral change, randomized controlled trial, overall survival

## Abstract

**Background:**

Despite the established benefits of regular exercise for patients with cancer to counteract the deleterious effects of the disease itself and treatment-related adverse effects, most of them do not engage in sufficient levels of physical activity and there is a paucity of data on the integration of efficacious exercise programs that are accessible and generalizable to a large proportion of patients with cancer into routine cancer care.

**Objective:**

We intend to examine the effects attributable to the implementation of a community-based exercise program on cardiorespiratory functional capacity and quality of life for patients with cancer.

**Methods:**

This will be a hybrid study. In the first experimental phase, patients diagnosed with any type of cancer will be randomized into two parallel groups. One group immediately performs Bizi Orain, a 3-month supervised exercise program (3 times a week), in addition to behavioral counseling in a primary health care setting; the other is a reference group that starts the exercise program 3 months later (delayed treatment). In the second observational phase, the entire cohort of participants will be followed-up for 5 years. Any person diagnosed with cancer in the previous 2 years is eligible for the program. The program evaluation involves the uptake, safety, adherence, and effectiveness assessed after completion of the program and with follow-ups at 3, 6, 12, 24, 36, 48, and 60 months. The primary outcomes of the experimental study, to be compared between groups, are improved physical function and quality of life, whereas overall survival is the main objective of the prospective study. To analyze the association between changes in physical activity levels and overall survival, longitudinal mixed-effects models will be used for repeated follow-up measures.

**Results:**

A total of 265 patients have been enrolled into the study since January 2019, with 42 patients from the hematology service and 223 from the oncology service.

**Conclusions:**

Bizi Orain is the first population-based exercise program in Spain that will offer more insight into the implementation of feasible, generalizable, and sustainable supportive care services involving structured exercise to extend survival of patients with cancer, improve their physical function and quality of life, and reverse the adverse effects of their disease and related treatments, thereby reducing the clinical burden.

**Trial Registration:**

ClinicalTrials.gov NCT03819595; http://clinicaltrials.gov/ct2/show/NCT03819595

**International Registered Report Identifier (IRRID):**

DERR1-10.2196/24835

## Introduction

Cancer is one of the diseases that causes major public health problems worldwide. The lifetime probability of developing any type of cancer in Spain is 49.9% in men and 32.2% in women [1]. Currently, increasing incidence rates and decreasing mortality rates due to advances in early detection and treatment have translated to a larger number of people living with cancer. This leads to increased costs of cancer care and a growing burden on health care systems in terms of medical management, cancer surveillance, and supportive care [2].

The disease itself and the treatments used lead to increases in psychosocial distress and depression, impaired cognitive function, increased levels of pain and fatigue, and a significant reduction in cardiorespiratory fitness and strength, as well as muscle (accelerated sarcopenia and cachexia processes) and bone mass losses, alongside increases in body fat. Consequently, patients with cancer have a poor quality of life and a greater risk of developing comorbidities [3].

It has been estimated that 30%-50% of cancer cases are preventable by reducing exposure to modifiable risk factors such as a sedentary lifestyle, poor diet, and excess body fat, and hence, these risk factors have become the target of the global strategy for cancer prevention [4,5]. A growing body of evidence has demonstrated that patients with cancer who achieve the minimum of 150 min·wk^-1^ of moderate or 75 min·wk^-1^ of vigorous physical activity (PA) levels recommended by the American College of Sports Medicine [6] and Australian Association for Exercise and Sport Science [7] experience ~27%-35% lower rates of cancer-related death and recurrence [8]. Current evidence from experimental studies indicates that exercise reduces tumor growth and cancer-specific mortality in a dose-dependent manner [6]. To target tumor intrinsic factors, moderate-to-high intensity endurance exercise is better than light exercise [9]. During exercise, the release of several systemic factors (ie, catecholamines and myokines), sympathetic activation, increased blood flow, shear stress, and increased temperature exert immediate stress on tumor metabolism and homeostasis. Following long-term training, such acute effects lead to reductions in systemic inflammation and oxidative stress, hormone-receptor binding, and adaptations in the modulation of circulating factors (ie, insulin, growth factors, and sex-steroid hormones); they also lead to intratumoral adaptations allowing improved blood perfusion, enhanced immunogenicity, and changes in gene expression and metabolism, which contribute to slower tumor progression and may reduce the ability of cancer cells to form tumors in distant tissues [9,10].

Further, resistance training is particularly important for patients with cancer experiencing loss of muscle mass (ie, sarcopenia or cachexia) during and following treatment [11,12]. This type of training is associated with a 33% lower risk of all-cause death after adjusting for potential confounders, including PA [13].

Despite the established benefits of exercise for patients with cancer and calls to include it as an integral part of cancer treatment, the chances of this information reaching patients are considerably reduced by the self-reported lack of knowledge among oncologists concerning how to properly promote and prescribe exercise [14,15] and paucity of translation strategies for integrating efficacious exercise programs into routine cancer care [16]. Unfortunately, only ~13% of patients with cancer meet international PA guidelines objectively measured with accelerometers [17].

Nonetheless, in recent years, community-based oncological exercise programs have been gaining prominence. LIVESTRONG at the Young Men’s Christian Association (YMCA) [18,19] is a community-based exercise program that has shown sustainable benefits in terms of physical function and self-perceived health. Our previous findings from the “Eficancer” study also indicate that exercising thrice a week during a 12-week individually tailored exercise program conducted at local health centers can improve quality of life and physical function, and these improvements are sustained over time [20].

Exercise program accessibility remains a challenge in implementing the international PA guidelines, but this challenge could be mitigated with strategies and pathways that connect patients with exercise-related resources and a well-designed behavioral intervention program to counteract the common barriers to exercise faced by patients with cancer during and after first-line treatments [21]. Further, the long-term effects on the PA levels, physical function, survival, and cost-effectiveness of large-scale community-based programs that are accessible and generalizable to a large proportion of patients with cancer (ie, administered in a standard supportive care setting) are unknown.

The main objectives of this study are as follows:

- to examine the effects attributable to an exercise program on physical function, and quality of life, and adverse effects compared to health habit prescriptions, and to explore whether these effects vary by cancer type, stage and treatments, age, or sex
- to analyze the association between the actual exercise dose (ie, number of sessions and exercise intensity, objectively measured PA level with accelerometers) and measured outcomes
- to assess the cost-effectiveness of the program

## Methods

Bizi Orain, which means “live now” in Basque language, is an evidence-based exercise program that adheres to the American College of Sports Medicine guidelines for cancer survivors [6] and is based on the “Life Now” exercise program for people with cancer delivered in Australia [22]. The program is administered by the Primary Care Research Unit of Bizkaia-Biocruces Bizkaia Research Institute (PCRUB-BBRI) and Deusto University and is delivered at a network of health centers equipped with Bizi Orain exercise laboratories integrated into the public health system of the Basque Country (Osakidetza).

### Program Design

**Figure 1:**
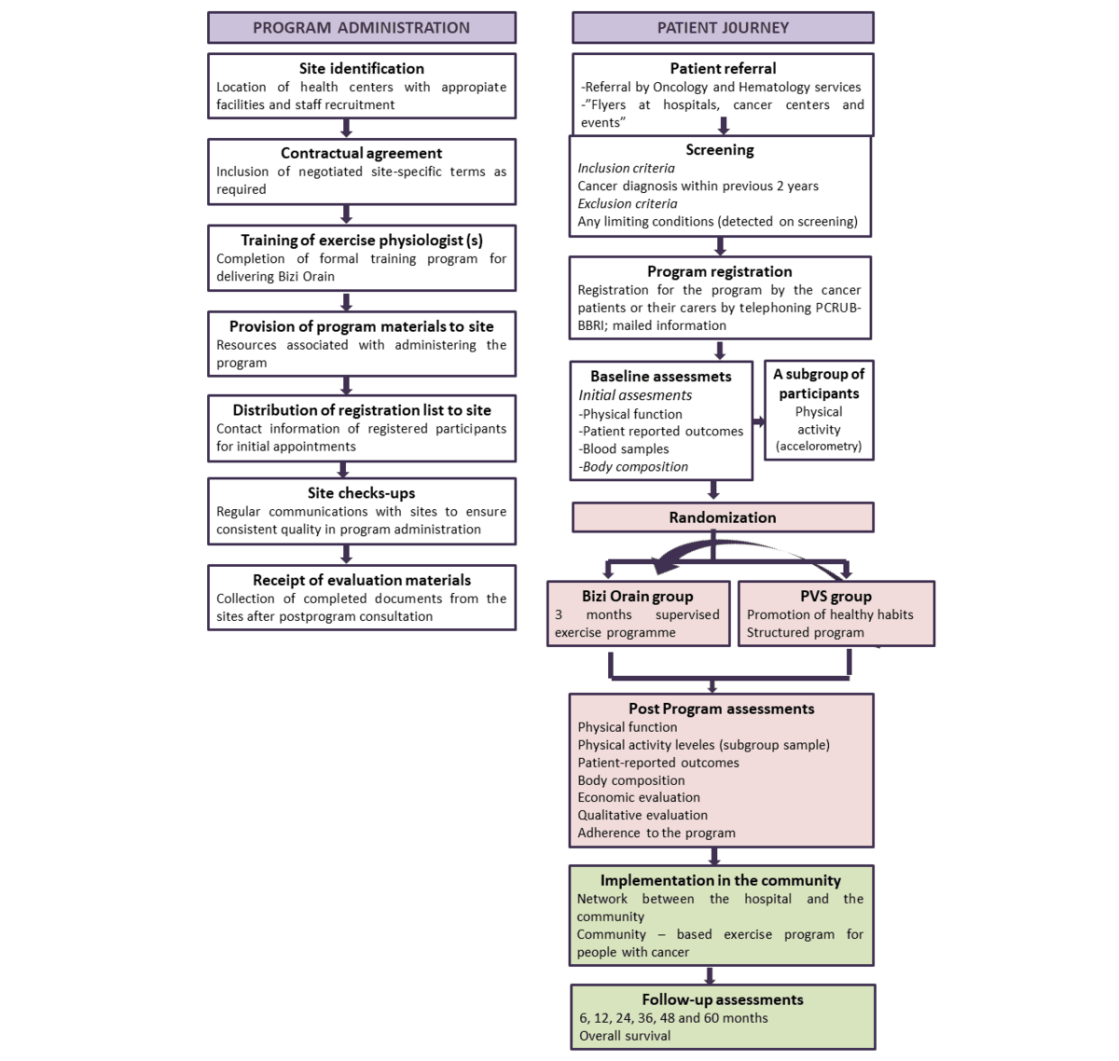
Schematic overview of the process for program implementation and evaluation.

Bizi Orain is a hybrid two-stage study. During the first 3 months, the study has a parallel-group randomized controlled clinical trial design, where study participants are randomly allocated (1:1) to either the Bizi Orain exercise program in addition to a previously reported behavioral intervention [23,24] for the promotion of healthy habits, or to the Prescribe Vida Saludable (PVS) group (In Spanish, “Prescribe Vida Saludable” means “prescription of healthy habits”) alone. After the postprogram assessments, participants allocated to the PVS group will initiate the Bizi Orain exercise program. Thereafter, a 5-year prospective observational cohort study will be conducted with follow-ups at 3, 6, 12, 24, 36, 48, and 60 months (Figure 1) to (1) examine the long-term clinical effects of PA exposure on overall survival, physical function, and patient-reported outcomes and (2) evaluate the feasibility of Bizi Orain, identifying potential barriers and facilitators for a generalized and sustainable exercise program within standard health care settings. To address this point, a qualitative research study will be conducted involving clinical, research, administrative, and community staff, and patients with cancer.

### Participants

Participant recruitment will last for 2 years starting from January 2019. People with any diagnosis of cancer currently receiving treatment or diagnosed less than 2 years earlier are eligible to participate and they are referred by their hospital oncologist, hematologist, or primary care general practitioner. To minimize the risk of hazards associated with program participation, patients will be excluded if they meet any of the following criteria: (1) neutropenia: absolute neutrophil count<500 mm^3^; (2) severe anemia: hemoglobin concentration<0.8g/dL; (3) platelet count<50000 μL; or (4) any musculoskeletal, cardiovascular, or neurological disorder that could place the participant at risk of injury or illness resulting from the exercise program (as determined by the patient’s clinician).

Numerous approaches are to be adopted to raise awareness about the program including the following: (1) training for oncology clinicians and support staff to facilitate direct referral of patients; (2) distribution of program flyers at hospitals, cancer centers, and community-based organizations as well as events for health professionals and patients; 3) sending information (by email and post) to people who have contacted the regional cancer association and expressed interest in exercise; 4) advertisements and coverage in local media; and 5) posts on the regional cancer association website and social media accounts. Specifically, the hospital’s Department of Oncology has established a system for identifying eligible patients. The oncologists, hematologists, or primary care general practitioners who have identified eligible patients inform them about the study, invite them to participate, and give them a written informed consent form. Once a patient agrees to participate, the clinicians inform the program administrators, and in the case of the hospital specialists, the patient’s general practitioner. Potential participants are able to self-enroll in Bizi Orain by telephoning the program administrators and by direct referral from their clinician. Eligible patients who agree to participate are invited to the baseline assessments. Participants are included in the study once they have signed the informed consent form and baseline data have been collected.

### Randomization

Patients will be centrally randomized by PCRUB-BBRI in a 1:1 ratio into the two study groups. A researcher who is independent of the organization responsible for managing the study will randomly allocate patients to groups using a computer-based random number generator.

### Evaluation

Evaluation of the Bizi Orain exercise program involves measuring the uptake, safety, adherence, and effectiveness of the program. These analyses incorporate elements of the reach, effectiveness, adoption, implementation, and maintenance (RE-AIM) framework [25]. Evaluation of the effectiveness of the program involves comparisons among the preprogram, postprogram, and follow-up assessments. The evaluation will be undertaken yearly from the beginning of the recruitment (2019) and will proceed until the target sample size is achieved.

#### Uptake

The proportion of people participating in the Bizi Orain exercise program out of all people with cancer in the province of Bizkaia (Basque Country) who are eligible will be reported as the participation rate. People with cancer who register for the program but do not commence their participation will also be reported. The representativeness of the participants will be determined by comparing their demographic and clinical characteristics to those of the people diagnosed with cancer in Bizkaia. Information about cancer diagnoses will be derived from the Basque Health System Registry (Osabide).

#### Safety

The incidence and severity of any adverse events (ie, falls, muscle strains) that occur during the health center–based sessions will be monitored and reported by the supervising exercise physiologist/nurse using program-specific documentation. Additionally, participants will be asked to self-report the incidence and severity of any adverse events they experience during health center–based sessions or home-based exercise using program-specific documentation.

#### Adherence

Attendance at health-center–based exercise sessions and the reason for any missed sessions will be tracked throughout the program. Adherence to the recommended amounts of PA for cancer survivors [26] is to be assessed by 7-day accelerometer recordings obtained from a randomly selected subset of the participants. Further, completion of assessments at preprogram and postprogram time points as well as follow-up questionnaires will be reported. Compliance with the Bizi Orain exercise program procedures by exercise physiologists/nurseries at each site will be monitored through evaluation of program documentation (ie, screening, assessment, and exercise prescription documents).

### Effectiveness: Primary Study Outcomes

#### Physical Function (Clinical)

Physical function is assessed by 400-m walk tests in a 20-m corridor, repeated chair stand tests (5 times), and handgrip dynamometry tests [27-29]. Each participant will perform a graded submaximal cardiopulmonary exercise test (CPET) on an electric braking cycle-ergometer (ergoline GmbH, ergoselect 4) in the laboratory of the University of Deusto in a controlled environment (temperature, ±21 ºC; relative humidity, 50%-55%; barometric pressure, ±720 mm Hg). Briefly, the test protocol involves an unloaded 5-min warm-up followed by 1-min stages with 10-W workload increments from an initial workload of 20 W [30]. Participants will be asked to maintain a steady cadence between 60 to 70 rpm. Gas exchange will be recorded breath by breath using a CPET machine (Geratherm Ergostik). The test is performed until confirmation of at least one of the following criteria: (1) The second ventilatory threshold or the so-called “respiratory compensation point” (RCP) is observed from the Wasserman figures (respiratory equivalents, and O_2_ and CO_2_ partial pressure changes). (2) The respiration exchange ratio (RER)>=1.05 and rating of perceived effort (RPE)>8 on the 0-10–point Borg scale [31]. (3) Participants exhibit volitional exhaustion without meeting the previous criteria.

From a pragmatic viewpoint and owing to the difficulties of performing a CPET in a community-based program setting, the results obtained from the CPET will not be used for individualized prescriptions of exercise intensity during the sessions. However, these results (ventilatory threshold heart rates) will be used to evaluate the actual exercise intensity undertaken by participants when following a standard exercise prescription based on simpler cost-effective methods like RPE and estimated heart rate reserve (HRR).

The maximal strength in the upper and lower body will be measured in terms of the 5-repetition maximum (5RM) (the maximum load that can be lifted five times) in chest and leg press exercises, respectively [32]. These assessments are to be conducted by an independent exercise physiologist not involved with administering the exercise intervention.

#### Patient-Reported Outcomes (Clinical)

A series of questionnaires with sound psychometric properties are to be used to assess general health and cancer-specific quality of life and cancer-related fatigue. The Medical Outcomes Study 36-Item Short-Form Health Survey (SF-36) is used to assess general health-related quality of life status across physical functioning, physical role functioning, bodily pain, general health, vitality, social functioning, emotional role functioning, and mental health domains (higher scores indicating a greater quality of life) [33]. Cancer-specific quality of life is evaluated by the core quality of life (QLQ-C-30) questionnaire developed by the European Organization for Research and Treatment of Cancer (EORTC) [34]. This questionnaire includes five functional domains (physical, role, cognitive, emotional, and social, with higher scores representing greater function/quality of life) and three symptom scales (fatigue, pain, and nausea, with lower scores indicating greater quality of life/less symptom severity). The General Health Questionnaire (GHQ-12) is administered to assess the psychological morbidity and possible psychiatric disorders [35]. Cancer-related fatigue is assessed using the Functional Assessment of Chronic Illness Therapy-Fatigue (FACIT-Fatigue) scale [36]. Finally, the Spanish version of the Alcohol Use Disorders Identification Test (AUDIT) is administered to screen for harmful alcohol consumption [37].

#### Overall Survival (Prospective Observations)

To assess survival, patients will be followed-up for 5 years. Overall survival will be measured from the time of randomization until death. Medical records and death certificates will be reviewed every year to obtain the survival status. The cause of death will be determined by reviewing medical and death records.

### Effectiveness: Secondary Study Outcomes

#### Anthropometry and Body Composition

The height will be measured using a wall stadiometer (Seca) and body composition with a bioimpedance analyzer (Inbody 770, In-Bldg). The participants will be seated in a quiet room for measuring the resting heart rate and blood pressure.

#### Blood Samples

A derivatives of reactive oxygen metabolites (d-ROMS) test will be performed to assess the levels of oxidative stress in the patients [38].

#### PA Levels

All patients randomly selected to have their PA assessed will be asked to wear an accelerometer (DynaPort MoveMonitor; McRoberts BV) for a full week. This will be fitted around the waist, with the sensor at the middle of the lower back, and should be worn throughout the waking hours, except during water-related activities (ie, swimming and showering). Further, patients are asked to complete a diary documenting the main PA they perform, how tired they feel, and how many hours they sleep each day. With these data, we will analyze the time spent performing moderate PA, time spent on sedentary activities, number of steps walked, and calories burned per day [39].

#### Health Economics

An economic evaluation will be performed in parallel with the trial to assess the health benefits, additional costs, and potential savings of including exercise therapy as a standard of care for patients with cancer. This health economics analysis will provide the relative value for money of exercise medicine compared with other health care interventions in this patient population, stratified by age, cancer type, point along the cancer continuum, and stage of the disease. Hospital resource usage and associated costs will also be assessed to compare the costs of secondary health care utilization between the intervention and reference (usual care+PVS) groups. All hospital events, including emergency department attendances and admissions, outpatient visits and procedures, and inpatient admissions for all causes will be explored to quantify and identify potential disease-related events, as well as the total health care resource use for all other purposes inclusive of comorbidities and other chronic diseases. The cost of providing the supervised exercise and PVS interventions will also be quantified. Health benefits will be measured in terms of the quality of life based on the SF-6D utility index derived from the SF-36 and converted to a health utility score to obtain quality-adjusted life years for cost-utility analysis [40].

#### Adverse Events

An external committee will review and compare all nonserious adverse events, whereas researchers will be obliged to report any serious adverse events to the research unit by email. A coordination committee with access to all the information it needs will undertake preliminary analyses of the data to monitor the safety of the program. This committee will be composed of individuals who are independent of the organization responsible for managing the study and members of the research team including the study coordinator; all are blind to patient allocation. In addition to serving on the committee, the coordinator will telephone the participating health centers daily to check on the progress of the study, report weekly on this progress to the principal investigator of the study, produce a monthly report with the study data, and provide recommendations to the study management team.

The trial was registered on January 18, 2019 (Clinical Trials.gov NCT03819595).The protocol has been approved by the local Clinical Research Ethics Committee (CEIC de Euskadi, PI2019016).

### Effectiveness: Additional Measures

#### Qualitative Evaluation

Knowledge generated by qualitative evaluation is essential for designing a tailored implementation strategy to address organizational and professional barriers that may hinder the adoption of the Bizi Orain program under routine conditions. The qualitative evaluation is designed around the use of focus groups to explore the perceptions of each population involved in the study: clinicians (oncology and hematology services), exercise instructors, community agents (local authorities, managers, and instructors of fitness centers), and patients. Briefly, 14 focus groups with 5-8 members each will be conducted to acquire a global and heterogeneous perspective of the Bizi Orain program: 1 for clinicians, 1 for exercise instructors, 6 for community agents (1 per exercise laboratory), and 6 for patients (1 per exercise laboratory). The moderation of the discussions is structured based on the Consolidated Framework for Implementation Research (CFIR) [41] and adapted to each population. This theoretical framework is a valuable instrument to detect and analyze barriers and facilitators. This framework differentiates 39 constructs, organized into 5 domains or dimensions, which influence the degree of the real implementation of a program or intervention. Specifically, the 5 domains of the CFIR are the following: “characteristics of the intervention,” “external context,” “internal context,” “characteristics of the individuals involved,” and “implementation process.” Owing to the need for intervention on contextual factors—a key element in the design of any implementation strategy—and promotion of cooperation between health organizations and the community, the CFIR adapts to the analytical needs of this evaluation. In addition, the research team will adopt an inductive perspective to favor the emergence of concepts and issues not covered by this theoretical framework, which are key to understanding the mechanisms of Bizi Orain. The discourse generated in each group will be audio-recorded and transcribed verbatim. Moreover, the moderator and observer will prepare notes during the fieldwork to complete and triangulate the recorded data.

Qualitative data analysis will be structured into three stages. First, a deductive analysis will be conducted to identify the constructs of the theoretical framework that influence the implementation of the Bizi Orain program. Second, an inductive analysis based on grounded theory [42] will be conducted to identify other emergent categories that have an impact on the program. Finally, a qualitative comparative analysis will be performed to identify the necessary and sufficient variables needed to improve the implementation of the Bizi Orain program in primary care exercise laboratories. Atlas Ti software (version 5.0, ATLAS.ti Scientific Software Development GmbH) will be used to analyze the qualitative data.

#### Exercise Intervention

The program operates throughout the year and is a free 12-week, small-group (~8 people) exercise program supervised by specially trained instructors. Participants are required to participate in supervised exercise twice a week and to exercise independently a third time, walking in the neighborhood of the health center at a given target exercise intensity. Carers of eligible participants are invited to attend the program with the care recipients.

#### Individual Consultations

Before commencing the program, each participant receives a one-on-one consultation with an exercise physiologist at Deusto University. This consultation involves screening of the health status and initial assessments to tailor the exercise prescription according to the type of cancer, stage and treatment history, severity of symptoms/adverse effects, comorbidities, PA habits, and personal preferences. Each participant’s exercise program is designed to provide optimal stimulus to the cardiorespiratory and neuromuscular systems while maximizing safety, compliance, and retention. Subsequently, patients return for further consultations to undergo assessments and assess their progress since initiating the program, discuss strategies to continue exercising after the program, and develop a plan to maintain long-term positive exercise behavior.

#### Group Exercise Sessions

**Figure 2:**
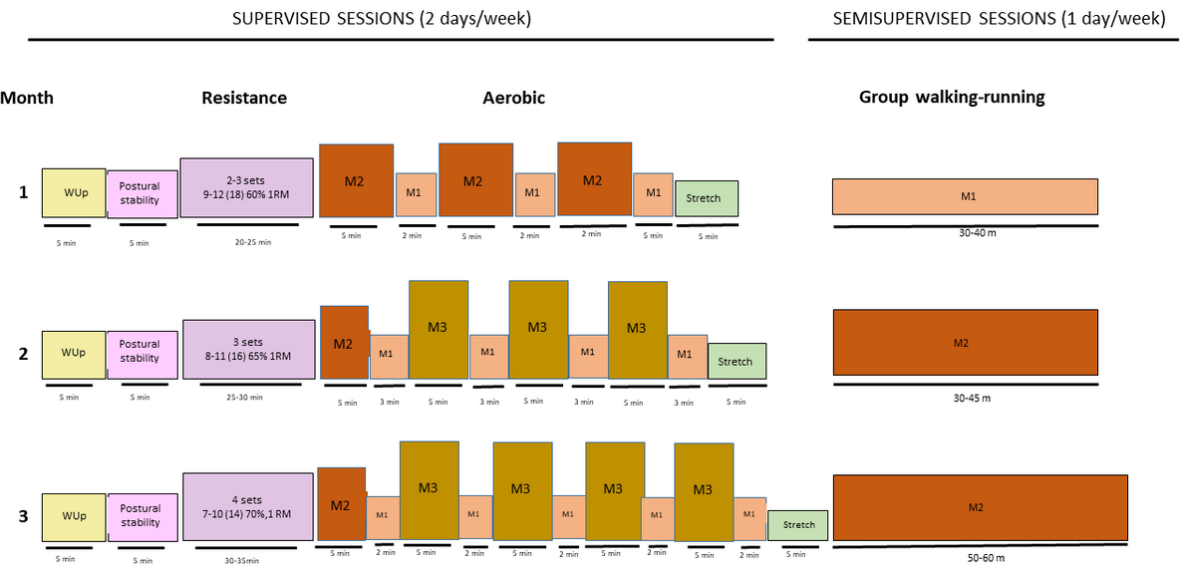
Bizi Orain exercise program. M1: Low-to-moderate intensity; M2: moderate intensity; M3: intensive moderate intensity; WUp: warm up.

Exercise sessions twice a week are conducted in groups of approximately 8 participants under the supervision of an accredited exercise physiologist or a nurse in the Bizi Orain exercise laboratories at the local health centers. The sessions last approximately 1-1¼ h and include a combination of moderate-to-high intensity aerobic and resistance exercises. The aerobic exercise component includes 30-35 min of at least moderate-intensity cardiovascular exercise using cycle ergometers and treadmills. The exercise intensity increases from moderate intensity for 8-min periods alternating with 2-min lower-intensity periods during the first month, moving toward higher-intensity 5-min intervals by the third month (Figure 2).

The exercise intensity zones are tailored to each patient by the estimated maximum heart rate using the equation 206.9 – (0.67 × age) [43] and applying specific intensity boundaries based on the HRR, defined as the difference between the resting heart rate and maximum heart rate. The %HRR has been adopted by the American College of Sports Medicine as the gold standard for indirect assessment of exercise intensity [44]. Every exercise session will be monitored with a heart rate monitor, teaching the patients to self-manage their exercise sessions with respect to the prescribed target intensities. The target intensity is between 40% and 85% of the HRR [44]. The perceived level of effort is recorded using the Borg RPE scale from 0 (rest) to 10 (maximal effort) [45], with the target intensity progressively increasing from 3 to 5-6 points (Table 1).

**Table 1:** Definitions of exercise intensity categories.

Zone^a^	%HRR^b^	RPE^c^	Training type
High-intensity training	>85	>5.5	High-intensity training. The patient nears exhaustion and is no longer in a steady state.
M3	60-84	4.5-5.5	Intensive moderate intensity. The patient has difficulties in talking and sweating increases.
M2	40-59	3-4	Moderate intensity. The patient notices an increased respiratory rate.
M1	20-39	1-2.5	Low-to-moderate intensity. The patient does not notice any increase in the respiratory rate.
Low-intensity training	<20	<1	Low intensity. This involves daily activities requiring low levels of effort.

^a^The intensity zones are based on the estimated maximum heart rate and applying specific intensity boundaries based on HRR [27]. RPE (determined using the Borg scale from 0 to 10) [28].

^b^HRR: heart rate reserve.

^c^RPE: rating of perceived effort.

The resistance component involves exercises that target six of the major upper and lower body muscle groups, as well as core exercises using exercise machines, bars, free weights, dumbbells, ankle weights, elastic bands, material for suspension training, and fitness balls. A progression from static exercises toward more dynamic exercises is encouraged, aiming to activate more muscle mass and thereby increase the cardiovascular demand. Among the main resistance exercise parameters that can be modified, the actual number of repetitions performed in a set, in relation to the maximum number that can be completed (ie, proximity to muscle failure), recently called “level of effort,” will be used to individualize the resistance exercise intensity, maximize the suitability of the exercise for each patient, and optimize the induced neuromuscular fatigue [46,47]. The target intensity is adjusted from 9 to 12 repetitions out of the 18 repetitions that could be completed (written as 9-12 (18)), which is equivalent to ~60% of 1 repetition maximum (1RM) using 2-3 sets during the first 4 weeks to 7-10 (14), which is ~70% of 1RM in the last 4 weeks of the program.

Participants undertake additional group-based walking (“park walking”) exercises, progressing from 30 min at low-to-moderate intensities to 40 min at moderate intensities. These walking sessions are self-managed by participants based on the perceived effort and heart rate. Patients’ carers are invited to attend these sessions. These strategies (ie, leadership, independence, and carer involvement) are based on the theory of planned behavior [48] and are designed to change attitudes toward exercise, increase perceived behavioral control, and influence the subjective norms or the social factors. We believe that if patients find these sessions are enjoyable, the long-term adherence will be higher, and this will have a significant impact on the long-term study outcomes.

### Behavioral Intervention (PVS)

PVS is a 5-step structured intervention delivered in routine primary care for the promotion of healthy habits [23,24]. It is based on strategies promoted by the United States National Cancer Institute and the counseling and intervention group of the United States Preventive Services Taskforce for the clinical management of PA, diet, and alcohol [48]. These strategies are considered the minimum effective health care interventions to produce behavior changes. The participant goes through a process of change structured around the five As (assess, advice, agree, assist, and arrange) construct for clinical counseling: (1) assessment of the participant’s PA, diet and smoking habits, beliefs, and attitudes, (2) provision of evidence-based advice and graphical information on the benefits and risks of these habits, as well as exploration of the participant’s intention to change these habits, (3) reaching an agreement for an appointment with the clinician to discuss the changes, (4) assistance in the design of an individualized plan that overcomes the barriers reported by the participant, and (5) arrangement for continued follow-up and modification of the plan.

**Statistical Analysis**

Data will be analyzed on an intention-to-treat basis with the maximum likelihood imputation of missing values, comparing average change scores at 3 months of follow-up (postintervention) among participants randomly assigned to the Bizi Orain or PVS groups. These analyses will include standard descriptive statistics, Student *t* tests, and analysis of covariance models adjusted for baseline values. These models will be extended to include clinically relevant covariates. Subgroup analyses will be conducted by cancer site and treatment status. Investigations into responders and nonresponders will be conducted to explore the heterogeneity of the intervention effect. To analyze the association between changes in the PA over time and outcome variables, longitudinal mixed-effects models will be used for repeated measures throughout the 5 years of follow-ups. Cox proportional hazards models will be used for survival analysis. No imputation method will be used to handle missing data as longitudinal mixed-effect models based on maximum likelihood estimation are more appropriate to handle missing data [49] than common imputation methods such as the last observation carried forward, complete case analysis, or other possible forms of imputation.

Finally, the incremental cost-effectiveness, cost-utility ratios, and confidence intervals will be calculated through bootstrapping and sensitivity analyses. All the analyses will be performed with SAS (version 9.4, SAS Institute) and R (version 3.6, R Core Team) statistical packages.

We have a network of 6 exercise laboratories with a capacity to include at least 1013 patients during the 2 years of recruitment. Assuming a 30% rate of loss (including deaths) throughout the year of follow-up, the study has a power of 80% to detect a difference in functional capacity between comparison groups, of at least 9 s in the 400-m walk test at 3 months as significant (α=.05), assuming a standard deviation of 95 m. Regarding the quality of life, the study has a power of over 90% to detect differences of 5 points between groups as significant (α=.05).

#### Quality Control

To ensure the quality of the study data, maximize the validity and reliability of the program, and accurate measurement of the variables, we will undertake the following steps:

- Produce documents for the study, including operational manuals for fieldwork and forms for registering measurements and details of the intervention.
- Store all documentation (informed consent forms, documents containing results, etc) in locked cabinets or on a secure server.
- Provide training for those responsible for the standardization of the study process, including specific training for nurses involved in the study, particularly for administration of the quality-of-life questionnaires.
- Hold regular meetings.
- Establish a coordinating committee and a data monitoring committee. As mentioned above, the coordinator contacts the health centers daily, requests information regarding the study progress, and reports to the principal investigator every week.
- Produce monthly progress reports.

### Ethical and Legal Aspects

This study protocol complies with the Declaration of Helsinki and its revisions, as well as with good clinical practice. The Ethics Committee of the Basque Country approved the study in the health centers ensuring it would be implemented in compliance with the established regulations. Regarding data confidentiality, only the study researchers have access to the data of individuals who agree to participate in the study, in compliance with the Organic Act 15/1999 of December 2013, on the protection of personal data and its 2011 revision.

## Results

A total of 123 patients have been enrolled into the study since January 2019, with 19 patients from the hematology service and 104 from the oncology service.

## Discussion

Bizi Orain seeks to substantially contribute to our knowledge concerning the effectiveness of an exercise program that is supervised and tailored for patients with cancer run in primary care centers under conditions of routine clinical practice.

Bizi Orain addresses these points through a multidisciplinary and innovative approach, applying evidence-based strategies [50] from behavioral counseling interventions [24,49,50] in primary care to promote sustained health and behavioral changes among patients and survivors of cancer. Specifically, the coordinating center at PCRUB-BBRI acts as a bridge between hospital-based and other referral pathways, and the health centers delivering the program. Patient recruitment is strengthened by providing training and progress reports for health care providers and other referral systems. The coordinating center has an extensive background in promoting and evaluating behavioral counseling interventions [24], with a multidisciplinary team of physicians, exercise physiologists, and therapists, specialized nurses, doctors in behavioral sciences, and statisticians, which provides a unique setting to assess, advise, assist, and evaluate a tailored exercise program for cancer survivors. In addition, the postprogram qualitative analysis will alert us to common concerns among the patients, and the main barriers and facilitators for providing effective and sustained exercise programs to cancer survivors.

Despite evidence-based guidelines [6,7] and a plethora of research demonstrating the benefits of exercise for patients and survivors of cancer, most patients do not receive clear instructions to exercise. Interaction with health care practitioners is a “window of opportunity” to increase PA engagement by patients and survivors of cancer; however, the strategy of simply making recommendations for PA seems not to fully capitalize on this opportunity. Although the common barriers to promoting PA among health care providers include lack of exercise-specific expertise and lack of time for exercise-related discussions amidst other clinical activities, health care providers need to be knowledgeable about the benefits of exercise during and after treatment, ensure patient safety by pre-exercise screening, and recommend and refer patients to existing community resources. Thus, the pathway to exercise as an adjuvant cancer therapy requires consideration of the following facilitators and barriers: (1) education for health care providers (about indications, guidelines, referrals, and safety) and integration of a qualified exercise professional into the clinical team, thereby reducing the burden on the health care providers (namely oncologists and oncology nurses), (2) educational handouts for patients about the benefits of exercise during and after treatment, as well as local access to focused programs for cancer exercise rehabilitation, and (3) self-management and behavior change skill development or resources for long-term exercise [51,52].

Although cancer exercise rehabilitation in hospitals is limited by the lack of resources, the lack of awareness about the potential benefits of exercise, and the lack of expertise within oncology units, several community-based programs have been reported and are ongoing [22,53].

Bizi Orain will add to our knowledge in clinical, epidemiological, and implementation fields by administering the program as a “real-world” intervention delivered in a standard supportive care service setting. The randomized controlled trial design of the study during the first 3 months will provide data on the impact of structured exercise on clinical outcomes. The large sample size allows for subgroup analysis, which may provide insight into how people with different cancer types and treatment statuses respond to exercise. Examination of the cost-effectiveness of the program represents a unique addition to the literature and significant advance in current knowledge regarding the potential value of cancer-specific exercise interventions to the health care system. The long follow-up period has the important goal of understanding the dose–response relationship between PA and mortality. Finally, qualitative analysis will help identify and overcome potential barriers toward developing a generalizable and sustainable exercise program as part of standard health care.

The major limitation of this study is possibly the length of the exercise program. It might not be the most appropriate design to evaluate the long-term benefits of regular exercise in terms of objectively measured and patient-reported outcomes and overall survival. On the other hand, Bizi Orain is an initial exercise program delivered in a standard health care setting that addresses patients’ needs during treatment or immediately after completion of the primary treatment, requiring greater supervision from qualified professionals owing to the number of adverse effects experienced at these times. Thus, delivering the program in a standard health care setting with qualified exercise physiologists, therapists, and nurses is appropriate. A second, community-based phase needs further investigation, but it could potentially contribute to maintaining higher PA levels, thereby reducing cancer-specific adverse effects and related comorbidities, resulting in a lower burden for health care systems, adding years to the lives of people with cancer, and most importantly adding quality of life to these years.

## Abbreviations

AUDIT: Alcohol Use Disorders Identification Test
CEIC: Clinical Research Ethics Committee
CFIR: Consolidated Framework for Implementation Research
CPET: cardiopulmonary exercise test
d-ROMS: derivatives of reactive oxygen metabolites
EORTC: European Organization for Research and Treatment of Cancer
GHQ-12: General Health Questionnaire
HRR: heart rate reserve
PA: physical activity
PCRUB-BBRI: Primary Care Research Unit of Bizkaia-Biocruces Bizkaia Research Institute
PVS: Prescribe Vida Saludable (It means “prescription if healthy habits” in Spanish.)
RCP: respiratory compensation point
RE-AIM: reach, effectiveness, adoption, implementation, and maintenance
RER: respiration exchange ratio
RM: repetition maximum
RPE: rating of perceived effort
SF-36: The Medical Outcomes Study 36-Item Short-Form Health Survey
YMCA: Young Men’s Christian Association
